# Clonal Hematopoiesis and Risk of Incident Pericarditis and Myocarditis in 2 US Biobank Cohorts

**DOI:** 10.1001/jamacardio.2026.0258

**Published:** 2026-03-18

**Authors:** Yash Pershad, Kun Zhao, J. Brett Heimlich, Alexander G. Bick

**Affiliations:** 1 Department of Medicine, Vanderbilt University Medical Center, Nashville, Tennessee

## Abstract

This cohort study explores the association between clonal hematopoiesis of indeterminate potential and risk of incident pericarditis and myocarditis among participants in 2 large US biobanks.

Clonal hematopoiesis of indeterminate potential (CHIP), the age-related expansion of blood cells harboring leukemia-associated variants, promotes cardiovascular disease through inflammatory pathways including the NLRP3 inflammasome and interleukin 1β (IL-1β).^1^ Schuermans et al^2^ reported an association between CHIP and increased myocarditis and pericarditis risk among UK Biobank participants. External replication is essential to establish CHIP as a clinically relevant risk factor. Therefore, we examined the association between CHIP and pericarditis and myocarditis in 2 large US biobank cohorts.

## Methods

The Vanderbilt University Medical Center (VUMC) Institutional Review Board approved this cohort study. We analyzed data for 2006 to 2025 from participants (aged 40-90 years) in the National Institutes of Health All of Us and VUMC BioVU biobanks. Participants provided informed consent. CHIP was ascertained from whole-genome sequencing as described previously.^3^ We excluded individuals with prior hematologic malignant neoplasms or missing data. Participants with prevalent myocarditis or pericarditis were excluded from respective time-to-event analyses. We followed the STROBE reporting guideline.

We used covariates (age, sex, genetic ancestry, and others detailed in Table 1) and outcome definitions identical to those used by Schuermans et al.^2^ Age-scaled Cox proportional hazards regression models assessed associations between CHIP and incident myocarditis or pericarditis. Outcomes were defined as first events identified through *ICD-10* diagnostic codes identical to Schuermans et al.^2^ Inverse variance-weighted meta-analysis (using fixed-effects models when *I*^2^ < 25% and random-effects models otherwise) was performed. Two-tailed *P* < .05 was significant. All analyses were performed using R, version 4.5.0 (R Project for Statistical Computing).

**Table 1.** Baseline Characteristics of VUMC BioVU and NIH All of Us Participants Stratified by CHIP Status^a^

Characteristic	NIH All of Us Cohort (n = 201 572)		VUMC BioVU Cohort (n = 160 012)	
	No CHIP (n = 190 933)	CHIP (n = 10 639)	No CHIP (n = 151 446)	CHIP (n = 8566)
Age, median (IQR), y	60.7 (52.4-69.0)	67.8 (57.2-78.4)	55.3 (45.2-65.0)	66.4 (59.1-74.3)
Sex				
Female	114 283 (59.9)	5965 (56.1)	83 429 (55.1)	4252 (49.6)
Male	76 650 (40.1)	4674 (43.9)	68 017 (44.9)	4314 (50.4)
European genetic ancestry	120 142 (62.9)	7667 (72.1)	125 571 (82.9)	7458 (87.1)
Cardiovascular risk factor				
Current smoker	82 411 (43.2)	5013 (47.1)	57 046 (37.7)	3221 (37.6)
Hypertension	13 995 (7.3)	979 (9.2)	22 565 (14.9)	1508 (17.6)
Antihypertensive medication use	13 391 (7.0)	902 (8.5)	21 409 (14.1)	1401 (16.4)
Total cholesterol, median (IQR), mg/dL	180 (164-196)	182 (162-202)	188 (165-212)	184 (160-208)
LDL cholesterol, median (IQR), mg/dL	105 (98-112)	108 (101-115)	103 (101-106)	103 (97-103)
Cholesterol-lowering medication use	72 554 (38.0)	4788 (45.0)	51 492 (34.0)	3769 (44.0)
BMI, median (IQR)	29.1 (22.3-35.9)	29.7 (22.2-37.2)	27.8 (19.9-35.7)	28.5 (21.6-35.4)
Type 2 diabetes	52 637 (27.6)	3132 (29.4)	30 063 (19.9)	1903 (22.2)
Inflammatory heart disease				
Incident myocarditis	473 (0.2)	27 (0.3)	445 (0.3)	29 (0.3)
Incident pericarditis	987 (0.5)	74 (0.7)	989 (0.7)	62 (0.7)
Cohort follow-up duration, y	2.6 (1.0-4.1)	3.3 (1.0-4.2)	5.3 (2.1-11.2)	3.6 (1.2-8.8)

Abbreviations: BMI, body mass index (calculated as weight in kilograms divided by height in meters squared); CHIP, clonal hematopoiesis of indeterminate potential; LDL, low-density lipoprotein; NIH, National Institutes of Health; VUMC, Vanderbilt University Medical Center.

SI conversion factor: To convert total cholesterol and LDL cholesterol to mmol/L, multiply values by 0.0259.

^a^ Unless noted otherwise, data are presented as the No. (%) of participants.

## Results

We analyzed 361 584 participants in the All of Us (n = 201 572; median age, 61.1 [IQR, 52.7-69.5] years; 59.7% female, 40.3% male) and BioVU (n = 160 012; median age, 55.9 [IQR, 45.9-65.5] years; 49.6% female, 50.4% male) cohorts (Table 1). CHIP was associated with incident pericarditis (Table 2), largely replicating the findings of Schuermans et al.^2^ Any CHIP was associated with 1.65-fold increased pericarditis risk (95% CI, 1.29-2.10; *I*^2^ = 0). Large CHIP (variant allele fraction ≥10%) had a similar point estimate (hazard ratio [HR], 1.66 [95% CI, 1.17-2.35]; *I*^2^ = 0). *DNMT3A* CHIP accounted for the association (HR, 1.88 [95% CI, 1.45-2.43]; *I*^2^ = 0), while *TET2* CHIP showed a directionally concordant association (1.35 [0.76-2.40]). The association between CHIP and pericarditis persisted after adjusting for incident myocardial infarction.

**Table 2.** Multivariable-Adjusted Associations of CHIP and Gene-Specific CHIP Subtypes With Myocarditis and Pericarditis After Meta-Analysis of the VUMC BioVU and NIH All of Us Cohorts

Outcome and exposure	No. of participants	No. of events	Adjusted HR (95% CI)	*P* value
**Pericarditis**				
Any CHIP				
All of Us cohort	9394	25	1.72 (1.14-2.59)	.01
BioVU cohort	8263	40	1.60 (1.16-2.22)	.005
Meta-analysis	17 657	65	1.65 (1.29-2.10)	<.001
Large CHIP				
All of Us cohort	6989	23	2.15 (1.40-3.30)	<.001
BioVU cohort	7562	39	1.71 (1.23-2.38)	.001
Meta-analysis	14 551	62	1.66 (1.17-2.35)	.004
*DNMT3A* CHIP				
All of Us cohort	4835	13	1.70 (0.98-2.97)	.06
BioVU cohort	3754	20	1.63 (1.04-2.56)	.03
Meta	8589	33	1.88 (1.45-2.43)	<.001
*TET2* CHIP				
All of Us cohort	1889	3	1.01 (0.32-3.16)	.98
BioVU cohort	1946	9	1.53 (0.79-2.96)	.21
Meta-analysis	3835	12	1.35 (0.76-2.40)	.31
**Myocarditis**				
Any CHIP				
All of Us cohort	9423	7	0.93 (0.43-1.99)	.85
BioVU cohort	8273	17	1.53 (0.92-2.52)	.10
Meta-analysis	17 696	24	1.29 (0.84-1.97)	.24
Large CHIP				
All of Us cohort	7013	3	0.54 (0.17-1.68)	.28
BioVU cohort	7573	16	1.56 (0.93-2.61)	.09
Meta-analysis	14 586	19	1.53 (0.92-2.54)	.10
*DNMT3A* CHIP				
All of Us cohort	4839	4	1.02 (0.38-2.75)	.97
BioVU cohort	3756	10	1.87 (0.99-3.53)	.05
Meta-analysis	8595	14	1.30 (0.81-2.09)	.28
*TET2* CHIP				
All of Us	1899	2	1.40 (0.35-5.66)	.64
BioVU	1950	1	0.37 (0.05-2.63)	.32
Meta-analysis	3849	3	0.69 (0.21-2.26)	.54

Abbreviations: CHIP, clonal hematopoiesis of indeterminate potential; HR, hazard ratio; NIH, National Institutes of Health; VUMC, Vanderbilt University Medical Center.

CHIP was not associated with myocarditis in our 2 cohorts, but our results were directionally concordant with those of Schuermans et al (Table 2).^2^ Point estimates for any CHIP (HR, 1.29 [95% CI, 0.84-1.97]), large CHIP (HR, 1.53 [0.92-2.54]), and *DNMT3A* CHIP (HR, 1.30 [0.81-2.09]) were positive but not significant. The HR for *TET2* CHIP was 0.69 (95% CI, 0.21-2.26), with limited sample size. There was moderate heterogeneity between cohorts (*I*^2^ = 37.1%-68.2%).

## Discussion

This study using 2 US biobanks with 361 584 participants replicated the association^2^ observed previously between CHIP and pericarditis. Consistent effect sizes across 3 biobanks varying in enrollment settings strengthen evidence for CHIP-mediated pericardial inflammation.^4^ The biological plausibility of CHIP promoting pericarditis is supported by evidence that CHIP-associated inflammatory pathways, particularly NLRP3 inflammasome activation and IL-1β upregulation, are central to pericarditis pathogenesis.^5^ In another study, IL-1β inhibitors effectively treated recurrent pericarditis.^6^ We and Schuermans et al^2^ found that *DNMT3A*—but not *TET2*—CHIP was associated with pericarditis, although this may simply reflect statistical power. Future research should investigate the mechanisms underlying this source of heterogeneity by CHIP driver gene and whether anti-inflammatory therapies demonstrate benefit in individuals with CHIP and high pericarditis risk.

Study limitations include limited statistical power for myocarditis, CHIP ascertainment from whole-genome rather than deep targeted sequencing, potential residual confounding, and reliance on diagnostic codes. Alternative study designs (eg, myocarditis case-control studies) are necessary to explore the association between CHIP and myocarditis.

Our findings suggest that CHIP is a reproducible risk factor for pericarditis. Targeting CHIP-associated inflammatory pathways may represent a therapeutic strategy for preventing or treating pericardial inflammation in individuals at risk.

## Notes

Supplement.
Data Sharing Statement
