# Supplementary material for: Clonal Hematopoiesis and Risk of Incident Pericarditis and Myocarditis in 2 US Biobank Cohorts
Source: JAMA Cardiol. 2026 Mar 18;11(5):476–8. doi: 10.1001/jamacardio.2026.0258 (PMC13000738; doi:10.1001/jamacardio.2026.0258)
Supplement: Supplement. — Data Sharing Statement [file jamacardiol-e260258-s001.pdf]

Data Sharing Statement

Pershad. Clonal Hematopoiesis and Risk of Incident Pericarditis and Myocarditis in 2 US Biobank Cohorts. *JAMA Cardiol*. Published March 18, 2026.
doi:10.1001/jamacardio.2026.0258

Data
Data available: Yes
Data types: Deidentified participant data
How to access data: For BioVU, deidentified patient data is available to researchers whose proposed use of the data has been approved with a signed data access agreement by contacting biovu@vumc.org. AllofUs data is available to approved researchers at https://www.researchallofus.org.
When available: With publication

Supporting Documents
Document types: None

Additional Information
Who can access the data: Researchers whose proposed use of the data has been approved
Types of analyses: for purposes of replicating the original analyses
Mechanisms of data availability: with a signed data access agreement
